# In Vitro Antithrombotic and Hypocholesterolemic Activities of Milk Fermented with Specific Strains of *Lactococcus lactis*

**DOI:** 10.3390/nu11092150

**Published:** 2019-09-09

**Authors:** Miguel Ángel Rendon-Rosales, María J. Torres-Llanez, Aarón F. González-Córdova, Adrián Hernández-Mendoza, Miguel A. Mazorra-Manzano, Belinda Vallejo-Cordoba

**Affiliations:** Centro de Investigación en Alimentación y Desarrollo A. C. (CIAD), Carretera Gustavo Enrique Astiazarán Rosas, No. 46 Col. La Victoria, Hermosillo 83304, Sonora, Mexico

**Keywords:** bioactive peptides, simulated gastrointestinal digestion, cholesterol micelles, thrombin inhibitory peptides

## Abstract

Milk fermented with specific lactic acid bacteria (LAB) was reported to be a rich source of metabolites, such as peptides with different biological activities that may have a positive effect on cardiovascular health. Thus, in this study, the antithrombotic and hypocholesterolemic activities of fermented milk with specific strains of *Lactococcus lactis* were investigated before and after exposure to a simulated gastrointestinal digestion (SGD) model. The inhibition of thrombin-induced fibrin polymerization (IC_50_ peptide concentration necessary to inhibit thrombin activity by 50%), anticoagulant activity, inhibition of micellar solubility of cholesterol and bile acid binding capacity of water soluble fractions (WSF) <3 kDa from fermented milk were evaluated. Results indicated that the WSF from fermented milk with Lc-572 showed antithrombotic (IC_50_ = 0.049 mg/mL) and hypocholesterolemic (55% inhibition of micellar solubility of cholesterol and 27% bile acid binding capacity) activities. Meanwhile, fermented milk with Lc-571 showed mainly antithrombotic activity (IC_50_ = 0.045 mg/mL). On the other hand, fermented milk with Lc-600 presented mainly hypocholesterolemic activity (31.4% inhibition of micellar solubility of and 70% bile acid binding capacity). Moreover, biological activities were not lost after simulated gastrointestinal digestion conditions. Thus, fermented milk with these specific *L. lactis* strains show potential for the development of functional foods.

## 1. Introduction

Atherosclerosis and thrombosis are key processes for the development of cardiovascular diseases (CVD). Several epidemiological studies have described that high levels of cholesterol and atherogenic lipoproteins are the main risk factor of CVD [1]. Different approaches have been used to decrease these diseases, such as cholesterol-lowering and antithrombotic agents. Despite the progress and success in the treatment of CVD with pharmacotherapy, currently the problem is significantly high [2,3]. In addition to side effects of medications (hepatic damage, bleeding and allergies), non-pharmacological alternatives without these limitations have been proposed [4]. In the last decades, several reports support the protective role of some food compounds against CVD [5]. Bioactive peptides have also been the target of research due to their multifunctional properties that show potential to mitigate some risk factors associated to CVD [6,7].

Dairy proteins have been recognized as an important source of bioactive peptides coupled to their nutritional value, in fact several of the peptides with potential cardioprotective effect have been identified from these proteins [7,8,9]. Hypocholesterolemic and antithrombotic peptides derived from milk have been scarcely investigated. Those reported were obtained by specific enzymatic hydrolysis, usually trypsin [10,11,12]. Nevertheless, lactic acid fermentation is one of the most advantageous strategies used to obtain bioactive metabolites such as peptides [13]. The generation of milk peptides by lactic acid bacteria (LAB) is an interesting alternative, owing to their large variety of enzymes in their cellular proteolytic system. The specificity of the enzymes in LAB can also release a wide variety of bioactive peptides [14,15,16]. In fact, multifunctional peptides derived from milk fermentation have been reported [17,18]. Under this approach the selection of LAB strains capable of releasing peptides with several functions is important [19].

It was previously reported that milk fermented with specific LAB can reduce cholesterol levels, however, it was evidenced that the effect was due to potentially probiotic LAB [20,21]. Until now, the role of hypocholesterolemic peptides in fermented milk has not been fully described. It was suggested that certain components from the aqueous extract (whey) of milk fermented by a mixture of strains could bind to bile salts [22]. Furthermore, the administration of fermented milk with a non-probiotic strain improved the lipid profile in a rat model [23]. In relation to antithrombotic activity, it was previously studied in peptides derived from the caseinomacropeptide and lactotransferrin [24,25], which acted inhibiting platelet aggregation. Recently, it was evidenced that LAB can release thrombin inhibitor peptides from casein using *Lactobacillus casei* [26]. In addition, a bioinformatics approach based on molecular docking indicated that caseins are an excellent source of antithrombotic peptides [11].

Moreover, when food is ingested, it is subjected to gastrointestinal conditions, specifically proteins and peptides that can be totally or partially hydrolyzed by proteases [27,28]. The final result of this process greatly influences peptide function, stability and activity in the intestinal tract; these may be determined by the degree of hydrolysis, molecular weight, charge and hydrophobicity of peptides [29,30]. Nevertheless, studies on hypocholesterolemic [22] or antithrombotic [26] peptides derived from dairy proteins did not evaluate these bioactivities after being subjected to gastrointestinal conditions. For bioactive peptides to exert their biological activity one of the key requirements to be met is the ability to resist gastrointestinal digestion. In fact, in vitro, simulated gastrointestinal digestion is an initial screening experiment that may be used to study the fate of bioactive peptides prior to exploration of their bioavailability and bioactivity in vivo [27,29,30].

Therefore, the objectives of this study were to investigate the antithrombotic and hypocholesterolemic activities of fermented milk with different specific strains of *Lactococcus lactis* before and after exposure to a simulated gastrointestinal digestion model.

## 2. Materials and Methods

### 2.1. Substrates and Chemicals

Cholesterol, cholic acid, human thrombin (EC: 3.4.21.5), human fibrinogen, O-Phthaldialdehyde reagent (OPA), pepsin (EC: 3.4.23.1), pancreatin (EC: 232-468-9), bile salts, α-amylase (EC: 3.2.1.1), lysozyme (EC: 3.2.1.17), sodium taurocholate, bile assay kit, heparin sodium salt, mucin, bovine serum albumin (BSA), glucoronic acid, trichloroacetic acid, L-leucine, cholestyramine, linoleic acid, trichloroacetic acid, galactose and glucosamine were purchased from Sigma-Aldrich Chemicals Co. (St. Louis, MO, USA). M17 broth, lactose and dextrose were purchased from DIFCO (Sparks, MD, USA). Thrombin time and pro-thrombin time kits were purchased from Wiener Lab (Rosario, Argentina). A cholesterol assay kit was purchased from RANDOX Laboratories (Crumlin, UK). The DC Lowry protein assay was purchased from Bio-Rad Laboratories (Hercules, CA, USA).

### 2.2. Bacterial Strains and Growth Conditions

Five strains of *Lactococcus (L.) lactis*; NRRL-B-505071 (Lc-571), NRRL-B-50572 (Lc-572), NRRL-B-50600 (Lc-600), NRRL-B-50598 (Lc-598) and NRRL-B-50599 (Lc-599), were obtained from the Dairy Laboratory Collection at Centro de Investigación en Alimentación y Desarrollo, A.C. (CIAD, A.C., Hermosillo, Sonora, México). Lc-571 and Lc-572 were activated (1% *v*/*v*) in M17 broth supplemented with lactose solution (10% *w*/*v*) and incubated for 24 h at 30 °C. The process was repeated twice under the same conditions [31]. Lc-598, Lc-599 and Lc-600 were activated in M17 broth with dextrose solution (10% *w*/*v*) for 24 and 30 °C. These strains were subsequently cultivated (1% *v*/*v*) in two successive steps and incubated for 5 and 4 h, respectively. To prepare the inoculum, all active cultures were individually inoculated (3% *v*/*v*) in sterile (110 °C/10 min) reconstituted nonfat milk (10% *w*/*v*) and incubated for 12 h at 30 °C.

### 2.3. Preparation of Fermented Milk 

Fermented milk was prepared as previously reported [31]. Briefly, the inoculum was cultivated (3% *v*/*v*) in pasteurized (80 °C/30 min) nonfat reconstituted milk (10% *w*/*v*). Milk was incubated for 24 or 48 h at 30 °C to obtain fermented milk with 24 or 48 h of fermentation. The fermentation process was stopped by applying heat treatment (75 °C/15 min) and subsequent cooling at 4 °C. The samples of fermented milk were stored at −20 °C until further analysis.

### 2.4. Simulated Gastrointestinal Digestion Model

Samples from fermented milk with *L. lactis* were digested using a simulated gastrointestinal digestion validated model previously reported [32]. In that study, the model was validated for studying milk macronutrient decomposition in a three-step in vitro process. Validation was carried out by monitoring macronutrient degradation and comparing that those results were consistent with human physiological values [32]. As reported, the system consisted of three stages, which simulated the mouth, the stomach and the intestine (duodenum) [32]. Briefly, 4.5 mL of each fermented milk was incubated with 6 mL of artificial saliva and incubated for 5 min (pH 6.8). Thereafter, 12 mL of gastric juice was added to the mixture and incubated for 120 min (pH 2.5). Then, 12 mL of pancreatic juice and 6 mL of bile juice were simultaneously added. The mixture was incubated for 120 min (pH 6.5). All mixtures during the digestion process were incubated at 37 °C in a shaker water bath with 55 rpm. After total digestion, the chyme was rapidly cooled and stored at −80 °C until further analysis.

### 2.5. Determination of Free Amino Groups

In order to determine proteolysis after the fermentation process and digestion, the amino groups were determined with the OPA method [33]. Fermented milk (2.5 mL) was mixed with 5 mL of trichloroacetic acid (TCA, 0.75 N) and 0.5 mL of bidistilled water. The mixture was vigorously stirred for 1 min and allowed to stand for 10 min at room temperature. Subsequently, the mixture was centrifuged (Thermo Scientific, Chelmsford, MA, USA) at 4696× *g* (4 °C, 40 min) and the supernatant was collected and filtered through a 0.45 μm syringe filter (Millex Millipore, Billerica, MA, USA). Detection of free amino groups was performed at 340 nm (SpectraMax M3, Molecular devices, Sunnyvale, CA, USA) after mixing 30 μL of TCA-peptidic extract and 600 μL of OPA reagent after 2 min incubation at room temperature.

### 2.6. Preparation of Water-Soluble Peptidic Fraction

Digested and undigested fermented milk were centrifuged (4696× *g*, 4 °C, 40 min) and the supernatants or crude extracts were collected. Crude extracts were fractionated using a stirred ultrafiltration cell (Model 8050, Amicon, Bedford, MA, USA) with a molecular exclusion membrane (Ultracell 3 kDa, Millipore, Billerica, MA, USA). The water-soluble fraction (WSF) <3 kDa was lyophilized with a freeze-dryer (Labconco, Kansas City, MO, USA). Protein content in the lyophilized WSF was determined with the DC protein assay based on the Lowry method. Bovine serum albumin was used as a standard protein (0.1–1.5 mg/mL).

### 2.7. Size Exclusion Chromatography (SEC)

WSF (100 mg/mL mobile phase) were analyzed by size exclusion chromatography in an AKTA PURE (GE Healthcare, Piscataway, NJ, USA) equipped with a gel filtration column Superdex Peptide GL 10/300 mm (GE Healthcare, Piscataway, NJ, USA). WSF (100 μL) separation was carried using isocratic elution with 50 mM phosphate buffer (pH 7.0) and 0.15 M NaCl with a flow rate of 0.60 mL/min during 60 min. Peptides were detected at 280 nm. For molecular weight estimation, three molecular weight markers were used as reference standards (aprotinin, 6.5 kDa; Vitamin B12, 1.4 kDa and tyrosine (163 Da). Peptide molecular weights were calculated from a calibration curve constructed from data of elution volumes and molecular weights of reference standards [34].

### 2.8. Determination of Antithrombotic Activity

#### 2.8.1. Inhibition of Thrombin Induced Fibrin Polymerization

The antithrombotic activity in vitro was determined using the turbidimetric method based on the polymerization of fibrin by thrombin enzyme [26]. Briefly, the lyophilized WSF of either digested or undigested fermented milk was dissolved in buffer A (50 mM TRIS-HCl, with 0.12 mM NaCl, pH 7.2) at a protein concentration of 1 mg/mL. The microplate reader was set at 37 °C, in the plate wells, 140 μL of fibrinogen dissolved in buffer A (0.1 % *w*/*v*) and 40 μL of sample or buffer A were added. The first absorbance measured was taken at 405 nm after 10 min of incubation (sample blank or control blank). To begin the fibrin polymerization reaction, 10 μL of thrombin (12 U/mL) were added in the plate wells. The mixture was incubated for 10 min and the absorbance was recorded again (sample or control). The percentages of inhibition were calculated with the following equation.

Percentage of inhibition = [(C − CB) − (S − SB)]/(C − CB) × 100(1)

where, C, CB, S and SB represent the absorbance of the control (WSF plus fibrinogen), the control blank (fibrinogen plus buffer A), the sample (control plus thrombin) and the sample blank (control blank plus thrombin), respectively. Thrombin inhibitory activity by WSF was also expressed as the peptide content (mg/mL) necessary to inhibit thrombin activity by 50% (IC_50_).

#### 2.8.2. In Vitro Anticoagulant Activity in Plasma

The thrombin time and pro-thrombin time test were studied for anticoagulant activity in decalcified citrated plasma. Plasma was separated from blood cells in conical tubes containing sodium citrate (3.8% *w*/*v*) as an anticoagulant by centrifuging at 2200× *g* for 15 min. For the anticoagulant test, the evaluations were performed using commercial kits by the methodology previously described [35]. Briefly, plasma (150 µL) was mixed with 50 µL of WSF, then, they were incubated for 2 min at 37 °C. Immediately, coagulation time for thrombin time was recorded after inducing clotting by adding 200 µL of the thrombin time reagent. For pro-thrombin time, plasma (75 µL) was mixed with 25 µL of WSF, then, they were incubated for 2 min at 37 °C. Immediately, coagulation time for pro-thrombin time was recorded after inducing clotting by adding 200 µL of the pro-thrombin time reagent. The results of thrombin time and pro-thrombin time were expressed as seconds of clot forming.

### 2.9. Determination of Hypocholesterolemic Activity

#### 2.9.1. Inhibition of Micellar Solubility of Cholesterol

Artificial micelles were used as a model to determine the hypocholesterolemic activity [36]. The lipids with final concentrations of 0.5 mM of cholesterol, 2.4 mM of phosphatidylcholine and 1 mM of linoleic acid were dissolved in methanol and dried under a stream of nitrogen. The final lipid mixture was suspended in 15 mM of sodium phosphate buffer (pH 7.2) with 132 mM of NaCl, containing 6.6 mM of sodium taurocholate salt. The suspension (1 mL) was sonicated for 20 min using an Aquasoner system (Aquasonic 50D, VWR Ultrasonic cleaner, San Jose, CA, USA) and the solution was incubated for 24 h at 37 °C. Thereafter, 50 mg of WSF from digested or undigested fermented milk or cholestyramine (positive control) were added to 1 mL of micellar solution and the mixture was sonicated once more for 2 min. Subsequently, the suspension was incubated for 2 h at 37 °C and then centrifuged at 1000× *g* for 10 min. The supernatant was recovered and filtered with a 0.22 μm syringe filter. The remaining cholesterol content was quantified using an enzymatic colorimetric assay kit. The percentage of inhibition was calculated according to the following equation.

Percentage of inhibition (%) = [C_0_ − C_S_/C_0_)] × 100 (2)

where C_0_ represents the cholesterol content of the micelles and C_S_ represents the cholesterol content remaining in the micelles with the samples.

#### 2.9.2. In Vitro Bile Acid-Binding Capacity

The in vitro bile acid binding capacity procedure was performed with a slight modification methodology [37]. Sodium taurocholate salt or cholic acid was individually mixed in phosphate buffer (0.1 mM, pH 7). On the other hand, 100 mg of lyophilized WSF were dissolved in 1 mL of assay buffer and 100 μL of the WSF solution were transferred to a 1.5 mL microtube. Then 900 μL of bile acid solution was individually added. The mixture was incubated at 37 °C for 2 h at 300 rpm (Eppendord, Termomixer R mixer, Bridgman, NY, USA). Afterwards, the mixture was centrifuged at 22,000 × *g* and the supernatant was transferred to a volumetric flask. Subsequently, 1 mL of buffer was added to the precipitate and it was centrifuged again. The supernatant was recovered and placed in a flask, this operation was repeated once more and the solution was taken to 10 mL. The final solution was then ultrafiltered using a 1000 Da cutoff membrane (Ultracell 1 kDa, Millipore, Billerica, MA, USA). The bile acid concentration was determined using a fluorimetric kit. A standard curve of sodium cholate was prepared (0–25 μg). The amount of bile acid in the filtered solution was the bile acid that was not bound by the WSF solution. Cholestyramine was used as the positive control and results were expressed as the percentage of bile acid binding relative to cholestyramine.

### 2.10. Statistical Analysis

A completely randomized design was used and the normality test was performed before the one-way analysis of variance (ANOVA) was carried out. The means were compared using a Tukey–Kramer test. In addition, a comparison with a student paired *t*-test was used to compare the effect of gastrointestinal digestion on bioactivities. For all statistical analysis, a 95% confidence was used. The analysis was performed using NCSS 2007 (NCSS Statistical Software, Kaysville, UT, USA).

## 3. Results and Discussion

### 3.1. Determination of Free Amino Groups in Fermented Milk

**Figure 1:**
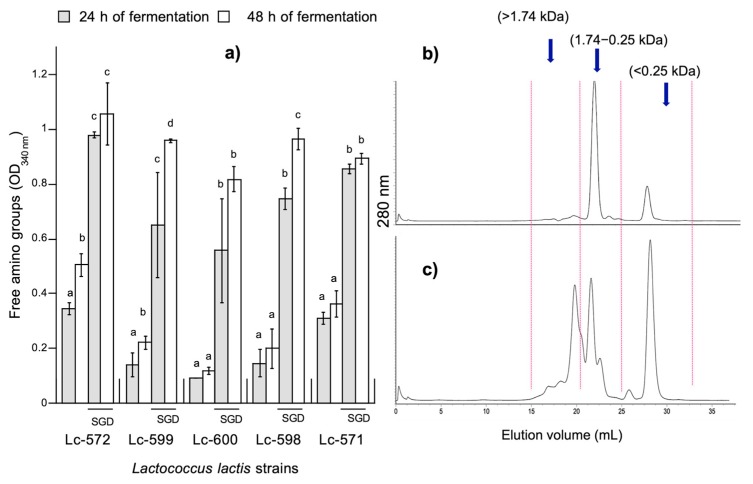
(**a**) Content of amino groups in fermented milk by *Lactococcus lactis* before and after simulated gastrointestinal digestion (SGD). Different letters (a–d) represent statistical differences (*p* < 0.05) between fermentation times and before and after SGD for the same strain. Data are the average (± SD) of triplicate analysis. Typical peptide profile of water soluble fractions (WSF) before (**b**) and after (**c**) SGD of fermented milk with *L. lactis*, obtained by size exclusion chromatography.

In order to know the degree of LAB proteolysis in the milk fermentation process and after digestion of fermented milk, the amino groups were quantified (Figure 1a). During fermentation, the amino groups increased when the fermentation time was increased from 24 to 48 h (*p* < 0.05). Lc-572 was the most proteolytic strain of all (*p* < 0.05), while Lc-600 was the least proteolytic (Figure 1a).

It was previously suggested that differences between capacities to hydrolyze proteins by LAB are due to the genotypic variation associated to their proteolytic system. These components include proteinases of the cellular envelope (PrtP), transporters and peptidases [14]. In contrast, strains with low proteolytic activity may present a higher capacity to biosynthesize amino acids for optimal growth, which do not depend on their proteolytic system. Moreover, wild strains develop a mechanism to be able to grow in adverse conditions [14,18]. This may be the case for fermented milk with Lc-600, which did not exhibit a high proteolytic activity.

When fermented milk was exposed to digestion, as expected, the concentration of amino groups was significantly increased (*p* < 0.05; Figure 1a). This indicated the extensive action of digestive proteases such as pepsin and trypsin against milk proteins or peptides derived from milk fermentation. In fact, a typical size exclusion chromatographic profile of fermented milk before digestion showed that fermented milk presented a large peak with a molecular weight in the range from 0.25 to 1.74 kDa and a smaller peak with molecular weight <0.25 kDa (Figure 1b). Furthermore, after digestion, fractions presented more peaks with a wider molecular weight distribution (Figure 1c). When LAB are used as a strategy for the production of bioactive peptides, it is important to consider proteolysis per se during milk fermentation; since they may release a greater amount of peptides, which may exert biological activity. Furthermore, these peptides derived from the fermentation process may continue to be hydrolyzed when ingested, to shorter peptides and amino acids, which may result in a loss or an increase in bioactivity [38]. Therefore, a simulated gastrointestinal digestion model is useful when peptides or other biological active compounds are evaluated [39].

### 3.2. Antithrombotic Activity

#### 3.2.1. Inhibition of Thrombin-Induced Fibrin Polymerization

**Figure 2:**
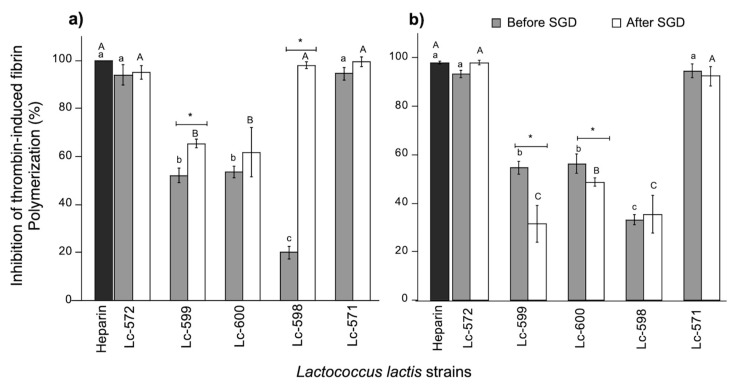
Inhibition (%) of thrombin-induced fibrin polymerization by WSF derived from fermented milk with 24 (**a**) and 48 h (**b**), before and after simulated gastrointestinal digestion (SGD). Different letters represent significant differences (*p* < 0.05) among strains before (lowercase, a–c) and after (uppercase, A–C) SGD. Asterisk (*) represents significant differences (*p* < 0.05) before and after SGD. Data are the average (± SD) of triplicate samples.

In atherosclerosis, the thrombosis process results from the activation of coagulation when there is endothelial damage, mainly derived from the rupture of atheromatous plaque [40]. Therefore, bioactive peptides capable to inhibit coagulation may prevent myocardial infarction [41]. In the present study, the inhibition of thrombin activity was evaluated as a mechanism to prevent the formation of clots. Figure 2 presents the inhibition percentages of thrombin-induced fibrin polymerization by WSF (1 mg/mL of protein) derived from fermented milk. The percentages were compared with heparin (1 mg/mL) as an anticoagulant. After proteolysis at 24 h of milk fermentation (Figure 2a), the results showed that there was thrombin inhibitory activity for all WSF.

The highest inhibitory (*p* < 0.05) activity was observed for WSF from fermented milk with Lc-571 or Lc-572. Likewise, WSF from fermented milk with Lc-571 or Lc-572 did not present significant differences with respect to heparin (*p* < 0.05). Furthermore, when fermentation time was increased to 48 h (Figure 2b), WSF from fermented milk with Lc-571 or Lc-572 maintained high inhibitory activities.

Interestingly, no significant differences (*p* > 0.05) were exhibited for WSF from fermented milk with Lc-571 or Lc-572 before and after digestion at both fermentation times (Figure 2a,b). In fact, no differences were exhibited between these WSF and heparin (*p* > 0.05). On the other hand, for the WSF from fermented milk with Lc-598 after 24 h of fermentation, thrombin inhibitory activity significantly (*p <* 0.05) increased after digestion and was not different (*p* > 0.05) from heparin. Furthermore, the IC_50_ of thrombin inhibition by WSF from undigested fermented milk with Lc-571 or Lc-572 were the lowest (*p* < 0.05) at both fermentation times, indicating that their peptidic fractions were more effective in inhibiting 50% of thrombin activity compared to all other WSF (Table 1).

When fermented milk with Lc-571 (24 h) or Lc-572 (48 h) was exposed to digestion, the IC_50_ significantly decreased (*p* < 0.05). In fact, after digestion, IC_50_ was the lowest (*p* < 0.05) for WSF from fermented milk with Lc-571 (24 h) or Lc-572 (48 h). On the other hand, although the WSF from fermented milk with Lc-598 (24 h) after digestion presented high thrombin inhibitory activity, this milk showed a high value for IC_50_ (0.91 mg/mL; Table 1), meaning that more peptidic nitrogen was required to inhibit 50% of the enzyme activity.

Thus, WSF derived from fermented milk with Lc-571 and Lc-572, presented the highest antithrombotic activities, since they presented the lowest IC_50_ values not only before but also after digestion. A similar study [26] reported 80.7% of thrombin inhibitory activity after milk fermentation by *Lactobacillus casei* at 27 h of fermentation, however, IC_50_ values and the effect of gastrointestinal digestion conditions on this activity was not reported.

Although IC_50_ values for thrombin inhibitory activity of dairy protein hydrolysates or peptides was also not reported, IC_50_ values for hydrolysates from other food protein sources, such as amaranth, were higher (IC_50_ = 0.20 mg/mL) [34] than values found in this study.

Even though thrombin presents a complex recognition mechanism, it has been described that the enzyme presents preferences for peptide sequences containing arginine, proline and a hydrophobic residue [11]. In fact, previous studies reported that some of the sequences found in WSF from fermented milk with Lc-571 or Lc-572 were rich in these amino acids and presented angiotensin converting enzyme inhibitory (ACEI) peptides [42]. One of the sequences (QEPVLGPVRGPFPIIV) produced in fermented milk with Lc-571 [42] was reported to have antithrombotic activity [11].

#### 3.2.2. Anticoagulant Activity in Decalcified Plasma

In order to evaluate the ability of WSF from fermented milk with *L. lactis* to modulate the different coagulation factors, thrombin time and pro-thrombin time assays were used to determine the common and extrinsic pathway of coagulation in plasma. In particular, thrombin time indicates the presence of inhibitors in the third phase of coagulation (common pathway), and measures the time required to form a clot after activation of coagulation by the addition of the thrombin enzyme. The pro-thrombin time assay includes the evaluation of factors of the extrinsic pathway through the activation of coagulation by the addition of a tissue factor to plasma [43].

In general, an increase of thrombin time was observed when the WSF was added to plasma compared with the negative control (23.0 s; Table 2). After digestion WSF derived from fermented milk with Lc-571 at 24 h was able to prolong the time of plasma coagulation thrombin time the longest (52.07 s), followed by the WSF from fermented milk with Lc-572 at 24 h (41.87 s; *p* < 0.05). With respect to pro-thrombin time, all WSF exhibited anticoagulant activity (*p* < 0.05) in comparison with plasma without WSF (18.9; Table 2). The highest value of anticoagulant activity by pro-thrombin time (43.85 s) was also exhibited by the WSF from fermented milk with Lc-571, followed by the WSF from fermented milk with Lc-572 at 48 h (36.5 s; *p* < 0.05). 

**Figure 3:**
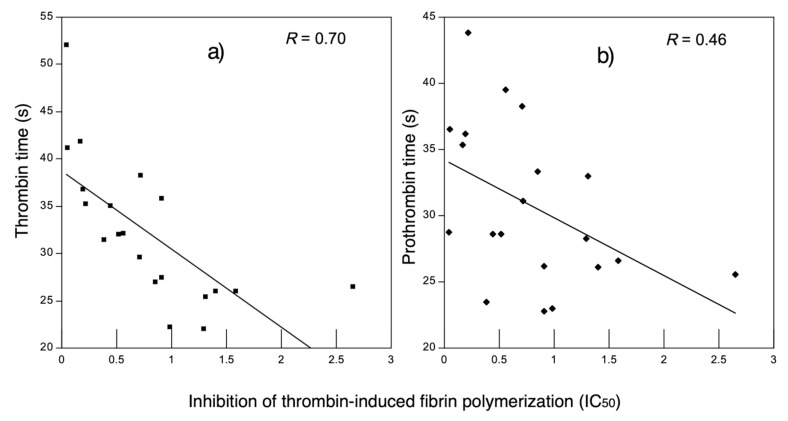
Correlation between thrombin-induced fibrin polymerization (IC_50_) and anticoagulant activity (**a**) thrombin time and (**b**) prothrombin time of WSF from fermented milk with *L. lactis*. A significant negative correlation (*p* < 0.05) was observed between the thrombin time (s) and thrombin inhibitory activity by WSF.

Correlation analysis between IC_50_ of thrombin-induced fibrin polymerization inhibition and thrombin time (Figure 3a) presented a significant negative association (*R* = −0.70, *p* < 0.05), indicating that at a lower IC_50,_ there was an increased clotting time in plasma. On the contrary, no significant correlation (*R* = −0.46, *p* > 0.05) was found between IC_50_ and pro-thrombin time (Figure 3b). These results may be explained by the fact that thrombin time evaluates thrombin-induced fibrin polymerization (common pathway). Therefore, this suggests that the WSF from fermented milk with Lc-571 or Lc-572 may contain specific peptidic fractions with thrombin inhibitory activity [11,42]. These results are in agreement with a previous study where a relation was observed between the thrombin time assay and the thrombin inhibitory activity but not with the pro-thrombin assay using amaranth protein hydrolysates [35]. Although inhibition (%) of thrombin-induced fibrin polymerization by WSF derived from some fermented milk was not significantly (*p* > 0.05) different from heparin, coagulation times for the WSF from fermented milks were significantly (*p* < 0.05) lower than those for heparin (>300 s). Heparin is used as a pharmacological treatment; however, fermented milks in this study are intended to be used as non-pharmacological treatment, since they may be used as a coadyuvants for the management of atherothrombosis.

Moreover, previous studies have reported that fermented milk with Lc-571 or Lc-572 showed ACEI peptides [42]. Their antihypertensive effect in an in vivo model with spontaneously hypertensive rats [23,31] and with prehypertensive subjects was also reported [44]. Those previous results and data presented in this study may suggest that fermented milk with Lc-572 or Lc-571 offer multifunctional cardioprotective properties.

### 3.3. Hypocholesterolemic Activity

#### 3.3.1. Micellar Solubility of Cholesterol

**Figure 4:**
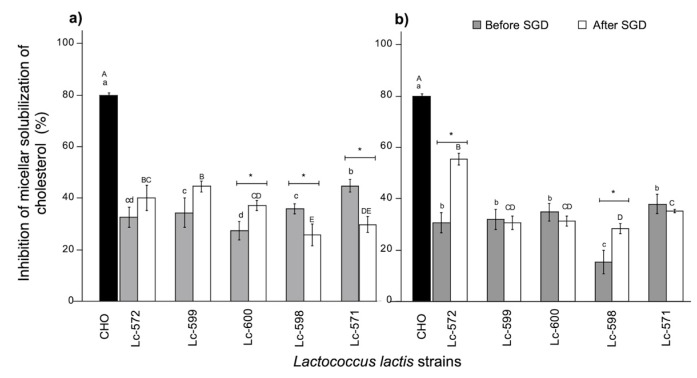
Inhibition of micellar solubility of cholesterol (%) by WSF from fermented milk with 24 (**a**) and 48 h (**b**) before and after simulated gastrointestinal digestion (SGD). Different letters represent significant differences (*p* < 0.05) among strains before (lowercase, a–d) and after (uppercase, A–E) of SGD for the same time. Asterisk (*) represents significant differences (*p* < 0.05) before and after SGD. Data are average (± SD) of triplicate samples. CHO: Cholestyramine.

Lipids such as cholesterol are insoluble in aqueous solution; therefore, to be absorbed in the duodenum and jejunum, they need to be emulsified in micellar aggregates containing bile salts, phosphatidylcholine, fatty acids and other compounds [45]. To investigate whether peptides were capable of preventing the formation of cholesterol micelles, the micellar solubility of cholesterol was studied. The results of inhibition of WSF or cholestyramine (positive control) are illustrated in Figure 4. At 24 h of fermentation, WSF from fermented milk with Lc-571 showed the highest inhibition of cholesterol micelles formation (*p* < 0.05) of all WSF before digestion (Figure 4a). Moreover, when the fermentation time was increased to 48 h (Figure 4b), the inhibition of cholesterol micelles formation was maintained (*p* > 0.05) for WSF with Lc-571. On the other hand, once fermented milk was exposed to digestion, the micellar inhibition increased significantly (*p* < 0.05) to 55.43% for the WSF from fermented milk with Lc-572 (Figure 4b).

To the best of our knowledge, there is no evidence that a peptidic fraction derived from milk fermentation may decrease cholesterol solubility in micelles. Furthermore, micellar cholesterol inhibition activity by the WSF from fermented milk with Lc-572 was higher than those reported for milk proteins (casein) hydrolyzed with commercial enzymes [10,45,46]. Due to these differences, it may be possible that the hypocholesterolemic activity of milk proteins may be improved by adding specific LAB, particularly Lc-572 strain. It has been reported that the hydrophobicity of peptides plays a major role in the hypocholesterolemic activity of peptides especially in binding bile acids [12]. The hydrophobic amino acids of the hypocholesterolemic peptides are thought to interact with bile acids by hydrophobic interactions leading to the formation of insoluble complexes [12]. Indeed, it has been reported that fermented milk with Lc-572 presented ACEI peptides [42] containing hydrophobic residues that may be responsible for the reduction of low-density lipoprotein cholesterol and triglyceride levels after fermented milk was administrated to spontaneously hypertensive rats [23].

#### 3.3.2. Bile Acid Binding Capacity

**Figure 5:**
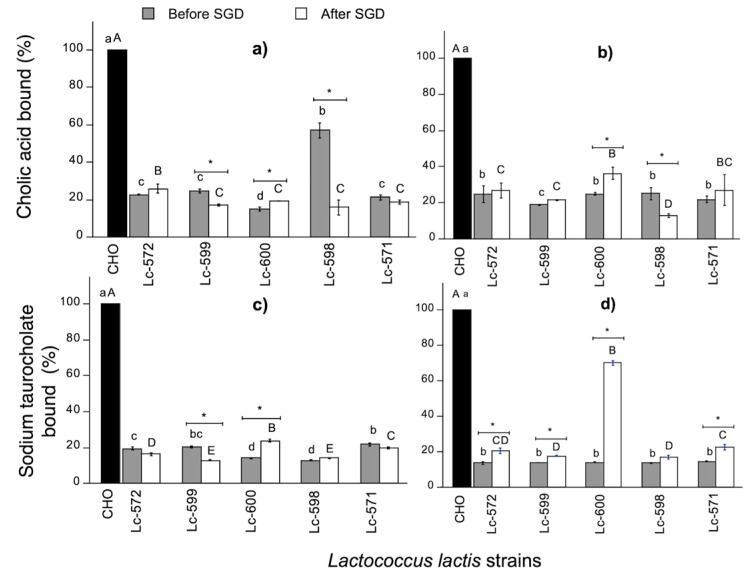
Cholic acid (**a**,**b**) and sodium taurocholate salt (**c**,**d**) binding (%) relative to cholestyramine by WSF from fermented milk with 24 and 48 h fermentation, before and after simulated gastrointestinal digestion (SGD). Different letters represent significant differences (*p* < 0.05) among strains before (lowercase, a–d) and after (uppercase, A–E) SGD for the same time. Asterisk (*) represents significant differences (*p* < 0.05) before and after SGD. Data are average (± SD) of triplicate samples. CHO: Cholestyramine.

Sodium taurocholate and cholic acid were tested individually and compared with cholestyramine. The binding of cholestyramine was established at 100% as reported by other studies [47]. Therefore, the binding capacity of WSF was expressed relative to the cholestyramine binding capacity. All WSF were capable of binding cholic acid (Figure 5a,b). At 24 h, the highest (*p* < 0.05) binding capacity was exhibited by the WSF derived from fermented milk with Lc-598 (57.2%) (Figure 5a). However, the binding capacity to cholic acid of this WSF significantly (*p* < 0.05) decreased to 16% after digestion (Figure 5a). On the other hand, fermented milk with Lc-600 significantly (*p* < 0.05) increased its binding capacity with sodium taurocholate to 70% after digestion (Figure 5d).

For the two bile acids tested, the WSF derived from fermented milk with Lc-600, showed the highest bile acid binding. However, taurocholate was bound two-fold more than cholic acid (Figure 5b,d). It was previously suggested that the binding capacity of peptides and hydrolysates is different depending on the bile acid type. The presence of a variable number of α-hydroxyl groups present in the bile acid structure may influence the association in the peptide-bile acid binding [12]. Other studies also reported binding capacities of protein hydrolysates to different bile acids [37]; however, the actual molecular interactions have not been elucidated.

Reports have investigated bile acid binding using fermented whey derived from nonfat milk using a mixture of *Lactobacillus* and *Streptococcus* and proteolytic enzymes (prozyme). The addition of proteases increased the binding capacity to bile acids [22]; however, their values (39%) were lower compared to those obtained in the present study (70%). In fact, casein hydrolysates using papain [48] and tryptic casein hydrolysates [10,49] showed a lower binding capacity in comparison to the results obtained in this study.

It has been reported that peptides with bile acid binding capacity, also present capacity to disintegrate cholesterol micelles [10,49]. However, these results did not show a positive significant correlation (*p* > 0.05) between both bile acid binding and inhibition of micellar solubility of cholesterol. This absence of positive correlation between micellar inhibition of cholesterol and bile acids may be due to the fact that peptides are specific for each mechanism [18,50]. Therefore, these results suggest that milk fermentation by Lc-572 or Lc-600 may be releasing peptidic fractions with different properties towards cholesterol-lowering activity.

## 4. Conclusions

The present study provides an in vitro evidence of the antithrombotic and cholesterol-lowering activities of peptidic fractions derived from milk fermentation by specific *L. lactis* strains. Fermented milk with Lc-572 presented antithrombotic and cholesterol-lowering activities, meanwhile, fermented milk with Lc-571 showed mainly antithrombotic activity. Previous studies showed that fermented milk with Lc-571 and Lc-572 presented peptidic fractions with an antihypertensive effect. Those findings in addition to results reported in this study, suggest that these fermented milks have potential for the development of functional fermented milk that may have a positive effect on cardiovascular health. In vivo studies are undergoing to confirm the antithrombotic and hypocholesterolemic activities of fermented milk with these specific *L. lactis* strains.

## Figures and Tables

**Table 1:** IC_50_ (mg/mL of peptide) values of inhibition of thrombin-induced fibrin polymerization by water soluble fraction (WSF) from fermented milk with *L. lactis* with 24 or 48 h of fermentation, before and after simulated gastrointestinal digestion (SGD).

WSF	24 h		48 h	
	Before	After	Before	After
Lc-572	0.19 ± 0.02 ^d^	0.17 ± 0.07 ^d^	0.71 ± 0.03 ^b^	0.049 ± 0.00 ^c,^*
Lc-599	1.29 ± 0.15 ^a^	0.38 ± 0.01 ^c,^*	0.91 ± 0.00 ^b^	>1.0
Lc-600	0.52 ± 0.02 ^c^	0.85 ± 0.01 ^b,^*	0.44 ± 0.032 ^c^	0.98 ± 0.0 ^a,^*
Lc-598	2.65 ± 0.26 ^a^	0.91 ± 0.04 ^a,^*	1.31 ± 0.17 ^a^	>1.0
Lc-571	0.56 ± 0.00 ^c^	0.045 ± 0.02 ^e,^*	0.45 ± 0.03 ^b^	0.22 ± 0.00 ^b,^*

All analytical results are the means (*n* = 3) ± standard deviation (SD). Literals (a–e) represent the statistical differences (*p* < 0.05) among strains for the same time of fermentation and conditions (before and after SGD). Asterisk (*) represents the statistical differences before and after SGD for the same fermentation time.

**Table 2:** Anticoagulant activity by thrombin time (s) and pro-thrombin time (s) on the plasma of water soluble fraction (WSF) from fermented milk with *L. lactis* with 24 or 48 h of fermentation, before and after simulated gastrointestinal digestion (SGD).

	Thrombin Time (s)				Pro-Thrombin Time (s)			
WSF	24 h		48 h		24 h		48 h	
	Before	After	Before	After	Before	After	Before	After
**Lc-572**	36.83 ± 0.17	41.87 ± 0.26 *	29.65 ± 0.54	41.17 ± 1.23	36.19 ± 0.97	35.33 ± 2.28	38.3 ± 3.11	36.5 ± 2.82
**Lc-599**	29.50 ± 0.48	31.50 ± 0.92	35.85 ± 0.32	26.00 ± 0.10 *	28.25 ± 0.35	23.5 ± 0.90 *	22.78 ± 1.71	26.61 ± 1.92 *
**Lc-600**	32.05 ± 0.23	27.00 ± 0.20	35.03 ± 1.02	22.2 ± 0.20 *	28.62 ± 0.02	33.25 ± 4.71	28.63 ± 2.68	22.98 ± 3.51 *
**Lc-598**	26.50 ± 0.23	27.50 ± 0.23	25.46 ± 0.43	26.00 ± 0.76	25.57 ± 0.79	26.17 ± 0.24	33.00 ± 0.70	26.13 ± 1.26 *
**Lc-571**	32.14 ± 0.56	52.07 ± 1.23 *	38.27 ± 1.20	35.27 ± 0.34	39.52 ± 1.44	28.74 ± 3.81 *	31.45 ± 0.70	43.85 ± 4.41 *
**NC**	23 ± 2				18.9 ± 0.56			

All analytical results are the means (*n* = 3) ± standard deviation (SD). Plasma without WSF was used as a negative control (NC). Asterisk (*) represents the statistical differences (*p* < 0.05) before and after SGD for the same time of fermentation (*t*-paired test).
